# Supplementary material for: Spatial determination and prognostic impact of the fibroblast transcriptome in pancreatic ductal adenocarcinoma
Source: eLife. 2023 Jun 23;12:e86125. doi: 10.7554/eLife.86125 (PMC10361717; doi:10.7554/eLife.86125)
Supplement: Supplementary file 1. — List of RNA probe panels used for Nanostring nCounter data collection including the immunoncology core panel, fibroblast-specific, housekeeping and negative control probe sets. [file elife-86125-supp1.docx]

**Supplementary Information**

| **Immunoncology core** | | | | **Fibroblast** | **Housekeeping** | **Negative** |
| --- | --- | --- | --- | --- | --- | --- |
| **L12B** | **IL6** | **CD11c** | **CXCR6** | **THY1** | **H3F3A** | **Neg Prb 1** |
| **IFNG** | **IFNGR1** | **CD11b** | **CTLA4** | **PDPN** | **SDHA** | **Neg Prb 2** |
| **STAT2** | **IFNAR1** | **4-1BB** | **CD40LG** | **CD34** | **POLR2A** | **Neg Prb 3** |
| **SDHA** | **ICAM1** | **TNF** | **PDL1** | **HLA-DRA** | **UBB** | **Neg Prb 4** |
| **PTEN** | **EPCAM** | **TIGIT** | **CD27** | **C3** | **RAB7A** | **Neg Prb 5** |
| **PECAM1** | **B2M** | **TBX21** | **BATF3** | **DKK3** |  | **Neg Prb 6** |
| **UBB** | **pan-Melanocyte** | **PDL2** | **VISTA** | **SFRP2** |  | **Neg Custom** |
| **OAZ1** | **Multi KRT** | **PD1** | **VEGFA** | **FAP** |  |  |
| **POLR2A** | **CXCL10** | **LAG3** | **STAT3** | **IL11** |  |  |
| **LY6E** | **CSF1R** | **IL15** | **STAT1** | **ACTA2** |  |  |
| **ITGB8** | **CD47** | **GZMB** | **CD45** | **OAZ1** |  |  |
| **ITGB2** | **CD44** | **FOXP3** | **PSMB10** |  |  |  |
| **ITGAV** | **CD40** | **DKK2** | **NKG7** |  |  |  |
| **CD20** | **CD8A** | **KI67** | **CD86** |  |  |  |
| **IDO1** | **CD68** | **ICOSLG** | **CD4** |  |  |  |
| **HLA-E** | **CD3E** | **HLA-DRB** | **B7-H3** |  |  |  |
| **HLA-DQA1** | **CCND1** | **HIF1A** | **CCL5** |  |  |  |
| **Tim3** | **BCL2** | **FAS** | **ARG1** |  |  |  |
| **CXCL9** | **AKT1** | **CTNNB1** | **RAB7A** |  |  |  |
| **CMKLR1** | **CD74** |  |  |  |  |  |

**Supplementary Table 1. NanoString nCounter** **RNA hybridisation probeset.** List of RNA probe panels used for Nanostring nCounter data collection including the immunoncology core panel, fibroblast-specific, housekeeping and negative control probe sets.
